# Development of a novel delivery quality assurance system based on simultaneous verification of dose distribution and binary multi-leaf collimator opening in helical tomotherapy

**DOI:** 10.1186/s13014-023-02366-6

**Published:** 2023-11-02

**Authors:** Yuichi Tanaka, Masatoshi Hashimoto, Minoru Ishigami, Masahiro Nakano, Tomoyuki Hasegawa

**Affiliations:** 1 Graduate School of Medical Sciences, Kitasato University, 1-15-1 Kitazato, Minami-ku, Sagamihara-shi, Kanagawa Japan; 2 School of Allied Health Sciences, Kitasato University, 1-15-1 Kitazato, Minami-ku, Sagamihara-shi, Kanagawa Japan; 3 Department of Radiology, Kitasato University Hospital, 1-15-1 Kitazato, Minami-ku, Sagamihara-shi, Kanagawa Japan; 4 Department of Radiation Oncology, Kitasato University School of Medicine, 1-15-1 Kitazato, Minami-ku, Sagamihara-shi, Kanagawa Japan

**Keywords:** Delivery quality assurance, Plastic scintillator, CCD camera, IMRT, Helical tomotherapy

## Abstract

**Background:**

Intensity-modulated radiation therapy (IMRT) requires delivery quality assurance (DQA) to ensure treatment accuracy and safety. Irradiation techniques such as helical tomotherapy (HT) have become increasingly complex, rendering conventional verification methods insufficient. This study aims to develop a novel DQA system to simultaneously verify dose distribution and multi-leaf collimator (MLC) opening during HT.

**Methods:**

We developed a prototype detector consisting of a cylindrical plastic scintillator (PS) and a cooled charge-coupled device (CCD) camera. Scintillation light was recorded using a CCD camera. A TomoHDA (Accuray Inc.) was used as the irradiation device. The characteristics of the developed system were evaluated based on the light intensity. The IMRT plan was irradiated onto the PS to record a moving image of the scintillation light. MLC opening and light distribution were obtained from the recorded images. To detect MLC opening, we placed a region of interest (ROI) on the image, corresponding to the leaf position, and analyzed the temporal change in the light intensity within each ROI. Corrections were made for light changes due to differences in the PS shape and irradiation position. The corrected light intensity was converted into the leaf opening time (LOT), and an MLC sinogram was constructed. The reconstructed MLC sinogram was compared with that calculated using the treatment planning system (TPS). Light distribution was obtained by integrating all frames obtained during IMRT irradiation. The light distribution was compared with the dose distribution calculated using the TPS.

**Results:**

The LOT and the light intensity followed a linear relationship. Owing to MLC movements, the sensitivity and specificity of the reconstructed sinogram exceeded 97%, with an LOT error of − 3.9 ± 7.8%. The light distribution pattern closely resembled that of the dose distribution. The average dose difference and the pass rate of gamma analysis with 3%/3 mm were 1.4 ± 0.2% and 99%, respectively.

**Conclusion:**

We developed a DQA system for simultaneous and accurate verification of both dose distribution and MLC opening during HT.

## Background

High-precision radiation therapies, such as intensity-modulated radiation therapy (IMRT) [1], volumetric modulated arc therapy (VMAT) [2], and helical tomotherapy (HT) [3, 4] are widely used. These irradiation methods require delivery quality assurance (DQA) to ensure treatment accuracy and safety. The conventional method for verifying IMRT treatment plans employs an ionization chamber [5], a film [6, 7], or a two-dimensional detector array [8–10]. These conventional methods have limited capability to measure specific points or planes, resulting in the interpolation of the calculated values for other points. Consequently, it may be challenging to detect dose errors at locations where actual measurements are not performed. To address this issue, frequent repositioning of the detector and performing multiple measurements are necessary; however, this approach is infeasible in clinical settings, where several plan verifications must be performed daily. Therefore, a verification approach based on three-dimensional (3D) dose distribution measurements is an effective solution to this problem. Several gel dosimeters have been developed for 3D dose measurement. However, the utilization of gel dosimeters has certain limitations, such as the time-consuming post-irradiation reading process via magnetic resonance imaging and the issue of reproducibility during phantom creation [11, 12]. Furthermore, because gel dosimeters are integrated dosimeters, they provide information pertaining only to the total dose and are not capable of real-time detection.

Recently, many studies have been conducted on IMRT verification, and various methods for measuring dose distribution using plastic scintillator (PS) emissions have been reported [13–18]. In these studies, a cooled charge-coupled device (CCD) camera or plenoptic camera was used to detect the scintillation light, which can be recorded using these cameras to obtain real-time information on the dose distribution. The PS is made using a water-equivalent material and affords the advantages of a linear dose response; it can be used as a phantom and can be easily fabricated into any shape. Hashimoto et al. [19] measured a binary multi-leaf collimator (MLC) operation using a PS and a general-purpose camcorder. Their study demonstrated the feasibility of using a simple device to measure complex instrumentation in HT. However, the camcorder employed in such a method had an 8-bit gradation, which was insufficient for measuring dose distributions. In addition, HT provides the purchasable option of real-time dosimetric measurement of all MLC movements utilizing the internal detector. However, this option can detect the movement of the MLC operation, the dose distribution cannot be measured simultaneously [20]. To address this limitation, we hypothesized that utilizing a CCD camera with 16-bit gradation would enable simultaneous measurement of mechanical motion and dose distribution, thereby allowing for a more detailed validation of the IMRT plan. Consequently, this study aims to develop a new DQA system capable of simultaneously verifying both the 3D dose distribution and MLC opening in HT, with such simultaneous capability being the major difference between existing DQA systems and the system proposed in this work.

## Material and methods

### Prototype detector

**Fig. 1:**
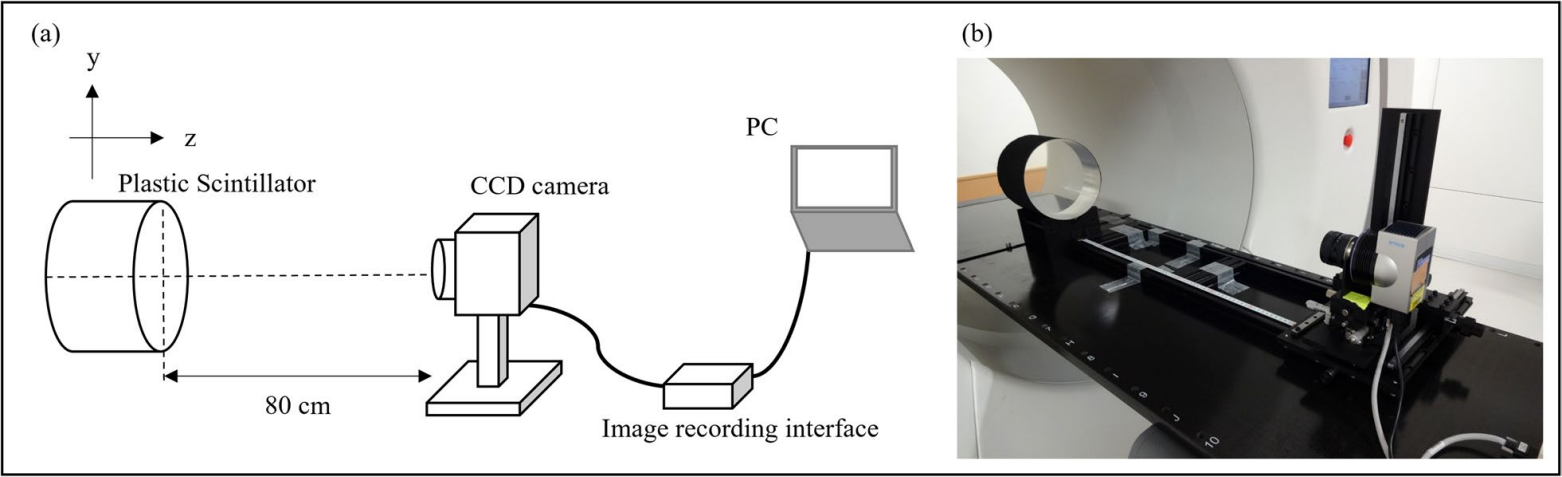
Measurement setup. **a** Schematic. **b** Photograph. A CCD camera was placed 80 cm away from the surface of the PS to collect the scintillation light. The images were recorded via an image recording interface to record as speedily as possible

We developed a prototype detector system consisting of a cylindrical PS (20 cm diameter and 15 cm thickness, BC-408, Saint-Gobain, Paris, France) and a cooled CCD camera (BU-51LN, BITLAN Corp., Gyoda, Saitama, Japan). The advantages of using PS as detectors include their short rise and decay times, and relatively easy machining. The short rise and decay times make it suitable for real-time measurements. In this study, we used a PS with a rise time of 0.9 ns and a decay time of 2.1 ns. The scintillation light generated by the plastic scintillator is reflected inside the HT gantry. To prevent the reflected light from entering the CCD camera, a light-shielding sheet is affixed to the side of the PS. This sheet was made of a material with minimal reflectivity. A CCD camera was placed to capture the entire PS and connected to a personal computer via an image-recording interface (BPU-30, BITLAN Corp., Gyoda, Saitama, Japan) (Fig. 1). The scintillation light was recorded using a CCD camera with a 16-bit grayscale of 512 × 680 pixels (1 pixel = 0.416 × 0.416 mm^2^). In this study, the aperture value of the lens (XN 0.95/25 CM120, Schneider Kreuznach, Germany) was determined to prevent saturation of the scintillation light in the widest irradiation field and was subsequently set to F1.3. The lens was focused on the center of the PS along the thickness direction. The frame rate used was 8.1 frames per second (fps), which was the fastest value for this CCD camera. The room was kept as dark as possible during the recording. Under these conditions, a CCD camera was used to record each scintillation light frame. As part of the preprocessing step, the background, defined as the average of 1000 frames of the unexposed images, was subtracted from the originally recorded images.

### Overview of measurement using our developed system

We used the TomoHDA system (Accuray Inc., Sunnyvale, CA, USA) for irradiation with 6 MV FFF beams, 860 MU/min dose rate, and a 2.5 cm fixed jaw. After aligning the center of the PS with the virtual isocenter of TomoHDA, the image center of the CCD camera was adjusted to align with that of the PS.

**Fig. 2:**
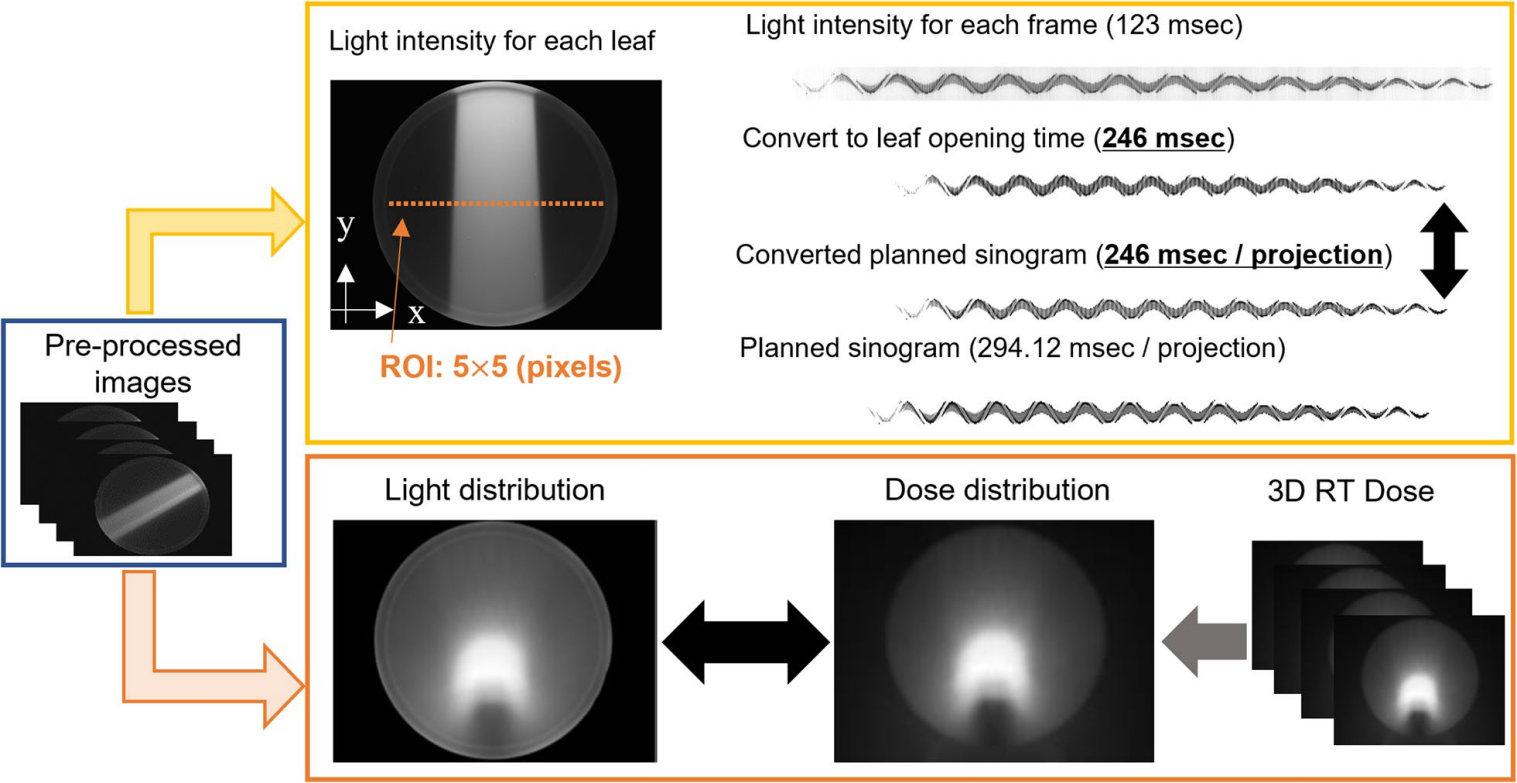
Analysis workflow. The recorded images are analyzed frame-by-frame to reconstruct a sinogram of the binary MLC, and the light distribution is obtained by integrating all the frames. The obtained measured values are compared with the sinogram, and dose distribution calculated by TPS, respectively

As shown in Fig. 2, the analysis workflow applied to the preprocessed images consisted of two approaches. First, ROI boxes measuring 5 × 5 pixels and corresponding to each MLC leaf were placed on the pre-processed images at a PS depth of 10 cm. While maintaining these ROIs static, we used gantry speed stability (introduced in the next section) to rotate the image in each frame to 0°. The MLC sinogram was reconstructed by measuring the average value of the pixels contained in the ROI of each frame. It entails detecting the on/off status of the MLC leaves in the measurement. The average pixel value is denoted as *q*_*n*_, where *n* indicates the number of MLC leaves. The measurements (relationship between the scintillation light and LOT, and field size dependency of the detected light) and corrections (lateral profile in the PS, irradiation time dependency and attenuation correction for the couch, changes in the detected light with the irradiation position in the depth direction) required for sinogram reconstruction are detailed in the next section. The reconstructed sinogram was then compared to the sinogram calculated through the TPS.

In the second approach, the light distribution was obtained by integrating the preprocessed images from all frames. The dose distribution calculated using the TPS was exported and added to the z-axis direction. For comparison, a point spread function (PSF) defined by a double Gaussian function (introduced in the next section) was used to correct the scattering of scintillation light in the PS. Finally, the obtained light distribution was compared with the dose distribution calculated by the TPS.

### Measurement of MLC opening

#### Relationship between the scintillation light and LOT

Investigation of the characteristics of the scintillation light detected by our system is necessary to measure MLC opening. Based on the procedure of Hashimoto et al. [19], we investigated the relationship between the scintillation light and LOT. The irradiation field was 10 cm (25–40th leaves opened), and the LOT varied from 29.41 to 294.12 ms for a gantry angle of 0°. The average light intensity in the ROI along the isocenter, 10 cm from the PS surface, was determined as *q*_center_ using Eq. (1):

1 $$\begin{array}{*{20}c} {q_{{{\text{center}}}} \left( {{\text{pixel}}\; {\text{value}}} \right) = \left( {q_{32} + q_{33} } \right)/2.} \\ \end{array}$$

#### Lateral (x-axis) profile in the PS

In this study, we used a cylindrical PS as the phantom, which is different from a conventional rectangular phantom. Therefore, acquiring a new lateral (x-axis) profile was necessary. We opened one leaf, irradiated it with X-rays for 294.12 ms from a gantry angle of 0°, and repeated the procedure for all the leaves from the 18th to the 47th leaf to measure *q*_n_.

#### Field size dependency of the detected light

In several irradiation fields (0.625–17.5 cm), profile measurements were performed at a LOT of 294.12 ms from a gantry angle of 0°. The *q*_*t*_ of each ROI was observed, and the scintillation light changes on the central axis and at the edge of the irradiated field were compared.

#### Irradiation time dependency and attenuation correction for the couch

The variation in X-ray output with irradiation time was examined during X-ray irradiation. The measurement conditions included an irradiation field of 10 cm and an irradiation time of 300 s. After X-ray irradiation, the *q*_center_ value was measured for each frame. An ionization chamber dosimeter (Standard Imaging, Inc., A1SL-A, Middleton, WI) and a potentiometer (Standard Imaging, Inc., TomoElectrometer, Middleton, WI) were used to compare the measurement results. The measurements were performed under the following conditions: an applied voltage of 300 V, irradiation field of 10 cm, and irradiation time of 300 s. During the measurements, the ionization chamber was placed at the center of the virtual water phantom at a depth of 10 cm. To investigate the degree of X-ray attenuation caused by the couch, measurements were performed when the gantry was rotated under similar irradiation conditions. The correction factor *k*_couch_ was determined by computing the ratio between the light intensity when the X-rays do not pass through the couch and that when the X-rays do pass through the couch.

#### Stability of gantry rotation speed

As HT involves rotational irradiation, we checked the stability of the rotational speed. The gantry rotation speeds were 15, 20, 40, and 60 s/rotation, and only the 32nd leaf was used for measurement.

#### Changes in the detected light with the irradiation position in the depth direction

In our system, the distance from the beam center to the CCD camera changes depending on the position where the PS is irradiated, causing the dimming of the scintillation light. Therefore, we investigated the change in scintillation light depending on the irradiation position. The measurement conditions were an irradiation field of 10 cm, and the couch was varied ± 5 cm along the z-axis from the center of the PS. The *q*_center_ at each measurement position in the z-axis direction was determined, and the ratio of *q*_center_ at the center of the PS was defined as *k*_depth_.

#### Reconstruction of MLC sinogram

A sinogram was reconstructed from the collected light *q*_*tn*_ (*t* is the number of frames) at each ROI, and the MLC opening was measured by comparison with the sinogram used during irradiation. Because the vertical axis of the sinogram shows the LOT per projection, converting the collected light *q*_*tn*_ to LOT was necessary. The relationship between the detected light and the LOT of the MLC, which was obtained from the characteristics of the scintillation light detected by our system, was used for conversion. In addition, the light intensity collected at each ROI varied owing to the use of a cylindrical PS and the movement of the gantry or couch. Therefore, the following equation was used:

2 $$\begin{array}{*{20}c} {q_{ctn} \left( {\text{pixel value}} \right) = q_{tn} \times k_{{{\text{lateral}}}} \times k_{{{\text{couch}}}} \times k_{{{\text{depth}}}} } \\ \end{array}$$

where* k*_lateral_ corrects for the difference in the light intensity collected in the nth ROI, *k*_couch_ corrects for the attenuation of X-rays due to the couch, and *k*_depth_ corrects for the scintillation light attenuation due to the irradiation position in the z-axis direction.

The cooled CCD camera collected data at a rate of 123 ms/frame, whereas irradiation was performed at a rate of 294.12 ms/projection. A direct comparison between the two rates is not feasible. To align the sinogram obtained through measurement closer with the sinogram used during irradiation, which had a duration of 246 ms, the measured sinogram was augmented with two additional frames. The sinograms used for irradiation were converted to a rate of 246 ms per projection using a C++ program that decomposed each projection into 1 ms segments and added every 246 decomposed segments. Because the ROI placed on the acquired image in Fig. 2 is fixed and rotating the ROI by gantry rotation is difficult, in this study, all images captured by the CCD camera were rotated at the PS center and returned to 0°. The gantry angles required to rotate images per frame were obtained from the gantry rotation speed.

During the X-ray irradiation of PS, scattered light and Cherenkov light may be generated, which can result in the collection of light, even in ROIs that correspond to closed leaves. A threshold value is established to mitigate these challenges. If *q*_*ctn*_ exceeds the threshold value, the leaf is considered “open”, and if *q*_*ctn*_ does not meet the threshold value, the leaf is considered "closed". Setting the threshold exceedingly high will result in incorrect identification of "open" leaves and setting it exceedingly low will result in the detection of incorrect and false signals. Therefore, the determination of the optimal threshold value is important to achieve an appropriate balance between the sensitivity and specificity of leaf opening/closing detection. Sensitivity is the percentage of open leaves that are correctly identified as open, and specificity is the percentage of closed leaves that are correctly identified as closed. The Youden index [21] was used to determine the threshold value, and the maximum Youden index was the optimal threshold value for determining the LOT, sensitivity, and specificity of detection. Simple pattern sinograms and IMRT sinograms were used to examine MLC opening. Here, the simple pattern sinogram shows only two states of the leaf (open or closed) within a single projection. An IMRT sinogram is an IMRT plan for simulating prostate cancer to measure the dose distribution.

### Measurement of dose distribution

#### Scattered correction of scintillation light

The scintillation light scattered in the PS resulted in a blurred distribution compared to the dose distribution calculated by the TPS. Therefore, we modeled the scattering component by combining a constant term and two Gaussian functions, as shown in the following equation:

3 $${\text{Post correction}} \left( {x, y} \right) = a \cdot \frac{1}{{2\pi \sigma_{1}^{2} }}e^{{\frac{{ - x^{2} + y^{2} }}{{2\sigma_{1}^{2} }}}} + b \cdot \frac{1}{{2\pi \sigma_{2}^{2} }}e^{{\frac{{ - x^{2} + y^{2} }}{{2\sigma_{2}^{2} }}}} + c,$$

where *σ*_*1*_ and *σ*_*2*_ are the standard deviations of the respective Gaussian functions, *a* and *b* are the proportions of the Gaussian functions, and *c* is a constant. To optimize the coefficients of the aforementioned equation, we compared the dose distribution of the scintillation light with that calculated using TPS. A conventional linear accelerator TrueBeam (Varian Medical Systems, Palo Alto, CA, USA) was used for X-ray irradiation with an energy of 6 MV, an irradiation field of 5 × 2.5 cm^2^, and a dose rate of 600 MU/min. The TPS was calculated on a 2 mm dose grid using Pinnacle3 (Philips Radiation Oncology Systems, Fitchburg, WI, USA).

#### Measurement of the IMRT plan

An IMRT plan (jaw size: 2.5 cm, gantry rotation speed: 15 s/rotation, pitch: 0.430) simulating prostate cancer was used to measure the dose distribution. The PS center and the isocenter were aligned during irradiation. The light distribution was obtained by integrating all frames of the pre-processed images collected during the irradiation of the IMRT plan. The obtained light distribution was compared with the dose distribution calculated using a Tomotherapy planning station (TPS; Accuray Inc., Sunnyvale, CA, USA). The comparison was based on two indices: dose difference (DD) and gamma analysis, utilizing a 3 mm/3% criterion and normalizing both results with respect to the maximum dose.

## Results

### Measurement of MLC opening

#### Relationship between the scintillation light and LOT

**Fig. 3:**
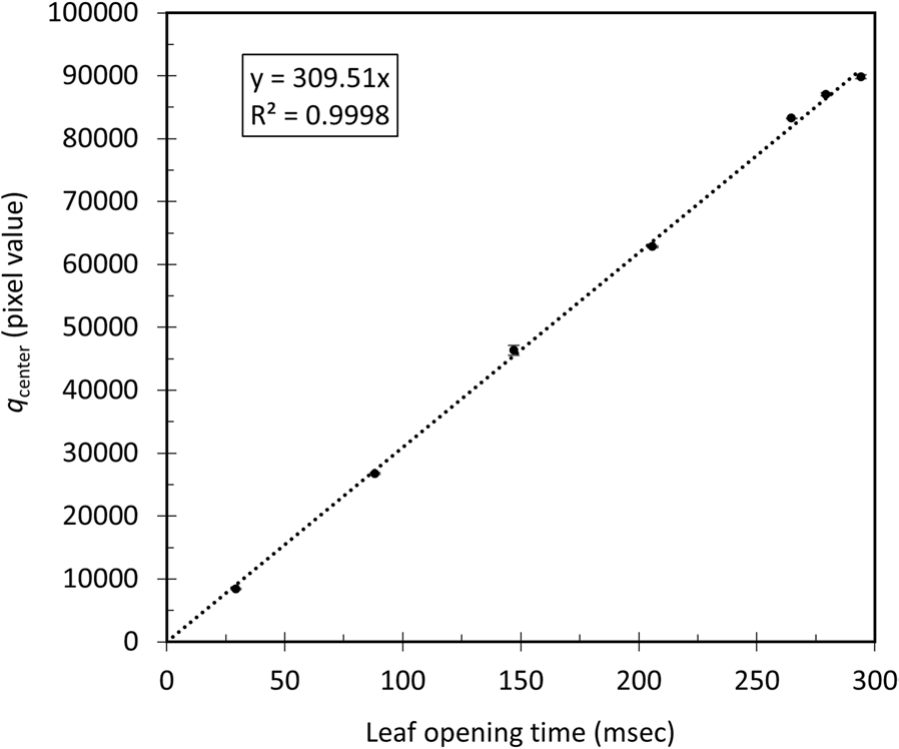
Relationship between the light intensity and LOT. Suitable linearity was observed from 29.4 to 294.12 ms

The relationship between the light intensity at the central beam axis and LOT is shown in Fig. 3. Suitable linearity was observed, and this relationship was used to convert the light intensity to LOT.

#### Lateral (x-axis) profile in the PS

**Fig. 4:**
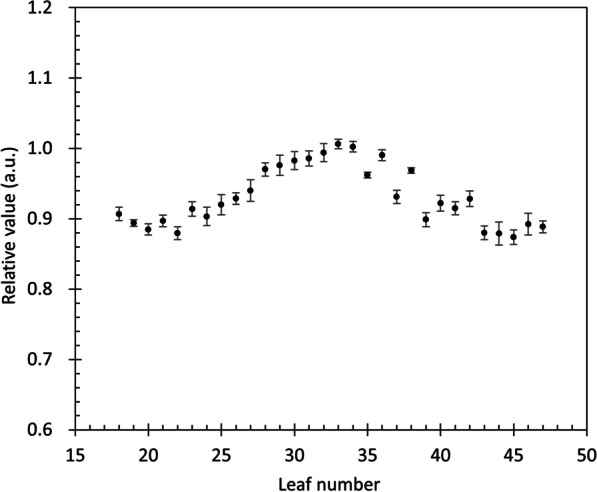
Amount of collected light at each ROI when each leaf was open sequentially. The amount of collected light in each ROI was normalized by the average value of the 32nd and 33rd ROIs corresponding to the isocenter

The light intensity obtained at each leaf was normalized by *q*_center_ and collected at a plane 10 cm deep at the isocenter; the results are shown in Fig. 4. The lateral profile peaked in the central leaf and decreased by approximately 10% at 10 cm to the left and right. This disparity primarily arises from variations in the depth dose at each ROI position. As for the asymmetry observed in the measured profiles, it can be attributed to leaf latency. The correction factor *k*_lateral_ was determined by considering the reciprocal of the normalized value for each leaf during sinogram reconstruction.

#### Field size dependency of the detected light

**Fig. 5:**
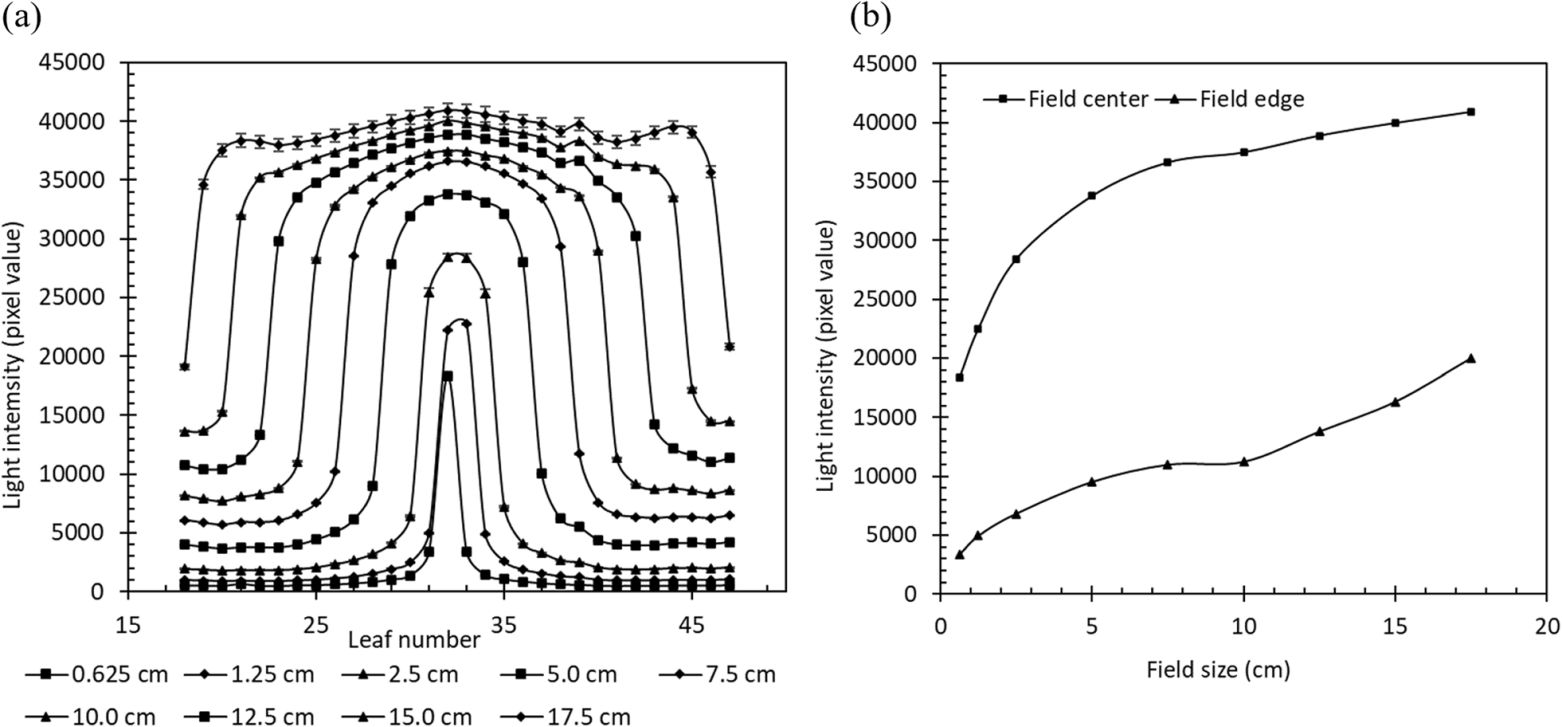
Field size dependency. **a** Relationship between the irradiation field size and the light intensity profile along the x-axis. **b** *q*_*t*_ at the center and the edge of the irradiation field for each irradiation field size

The relationship between the irradiation field size and the light intensity profile along the x-axis is shown in Fig. 5a. The *q*_*t*_ value for each ROI increased as the irradiation field size increased, probably owing to the scattering of scintillation light by the PS and Cherenkov light. The asymmetry observed in Fig. 5a for field sizes exceeding 12.5 cm is due to the staining of the PS surface of the 39th leaf. Figure 5b shows the *q*_*t*_ at the center and edge of the irradiation field for each irradiation field size shown in Fig. 5a. The center was at the 32nd leaf when one leaf was open, and between the 32nd and 33rd leaves when two or more leaves were open. *q*_*t*_ at the edge of the irradiated field was defined as the average of the adjacent outer leaves at both ends of the open leaf. For example, the end of the irradiation field when the 30th to 35th leaves were open was the average of *q*_29_ and *q*_36_.

#### Irradiation time dependency and attenuation correction for couch

**Fig. 6:**
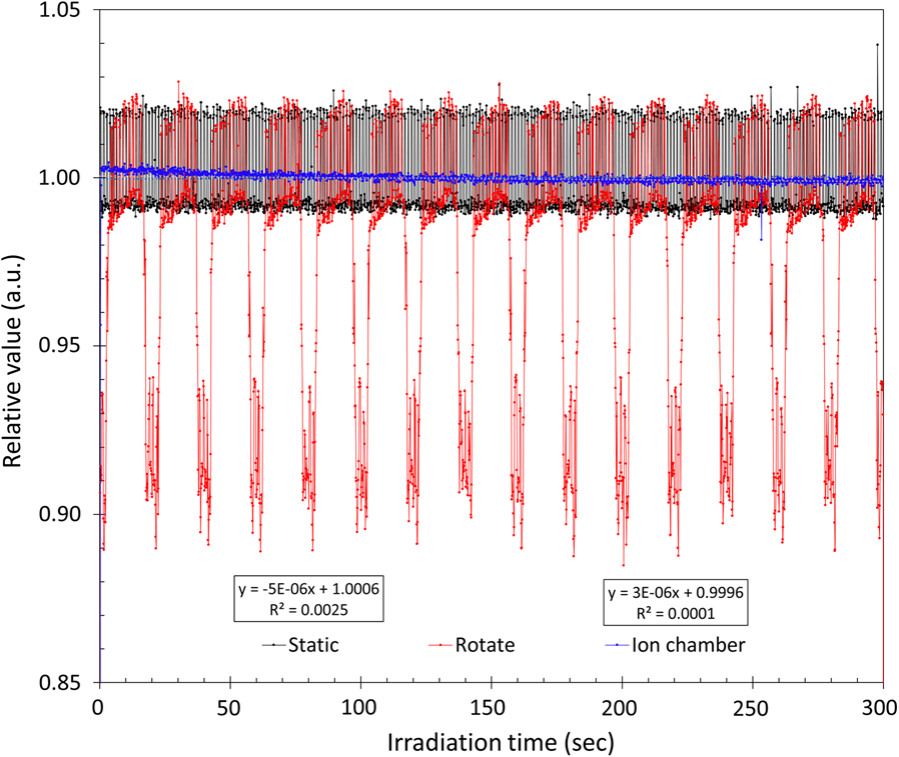
Relationship between the scintillation light and exposure time in the measured values of the ionization chamber. The black line indicates the scintillation light (gantry stationary), the red line indicates the scintillation light (gantry rotating), and the blue line indicates the ionization chamber dosimeter

Figure 6 shows the relationship between the irradiation time, the light intensity collected, and the values measured by the ionization chamber. The approximate curves for the collected light intensity and the ionization chamber dosimeters show $y=-4\times {10}^{-6x}+1.0005$ for the collected light intensity and $y=-1\times {10}^{-5x}+1.0014$ for the ionization chamber dosimeters, indicating a slight downward trend for both. The collected light intensity decreased by approximately 0.4% at 300 s, and the ionization box dosimetry value decreased by approximately 0.3%. Corrections were not made to reduce the light intensity. By comparing the static light intensity in Fig. 6 with the light intensity during rotational irradiation, the correction factor *k*_couch_ was used for sinogram reconstruction.

#### Stability of gantry rotation speed

**Fig. 7:**
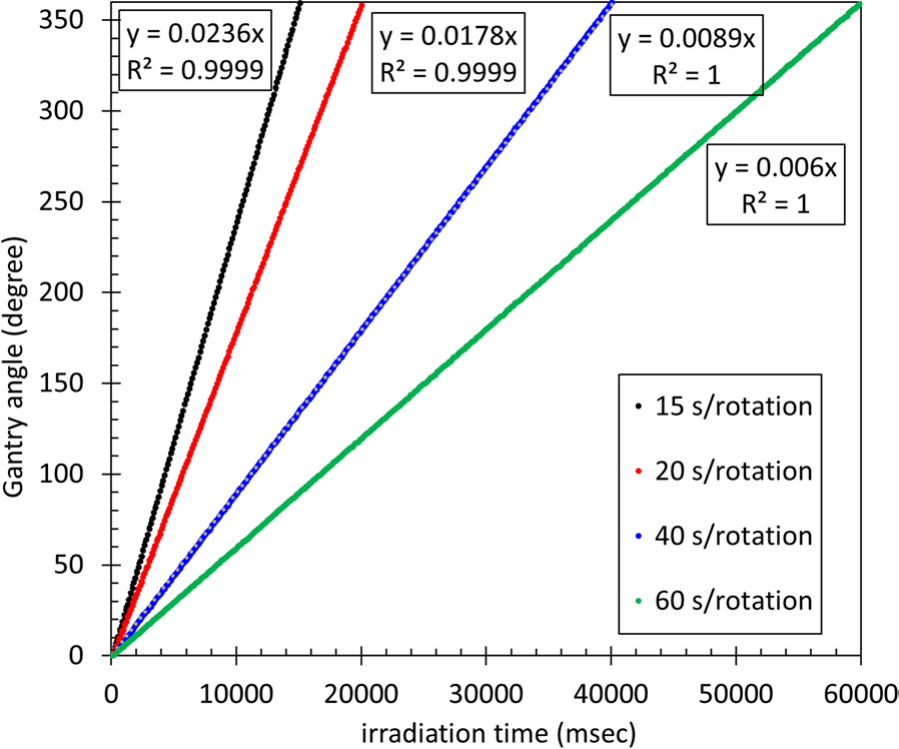
Gantry rotation stability. The solid line shows the theoretical value when the gantry rotation speed is assumed to be constant, and the dashed line shows the measured value

Figure 7 shows the gantry angle and the time from the starting position of the gantry rotation. The solid lines in Fig. 7 show theoretical values based on the assumption that the gantry rotation speed was constant, with an average error of 2.2% between the measured and theoretical values for rotation speeds from 15 to 60 s/rotation. This result indicates that the frame in which the X-ray beam is covered by the couch can be determined using the gantry rotation speed.

#### Changes in the detected light with the irradiation position in the depth direction

**Fig. 8:**
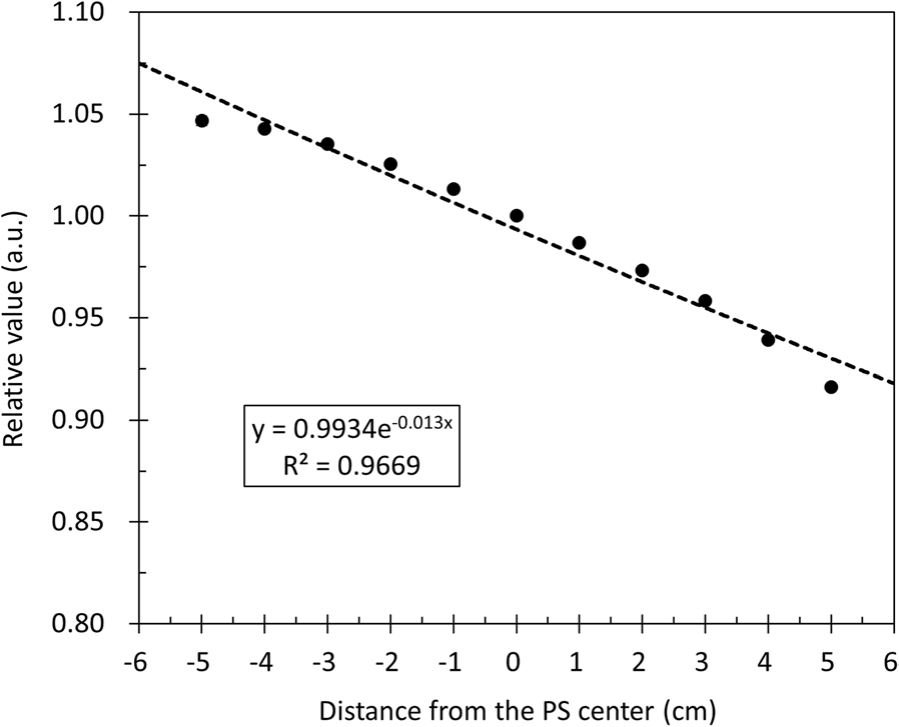
Relationship between the amount of detected light and the irradiation position within ± 5 cm in the z-axis direction from the beam center and the scintillation center. The vertical axis shows the normalized value at the center of the PS

The relationship between the irradiation position and the light intensity collected is shown in Fig. 8. A displacement of ± 5 cm in the z-axis direction results in a slight change of approximately 10%. An exponential function was used to approximate the measured value, which can be expressed as $y=0.9976\times {e}^{-0.011x}$. To compensate for the change in light due to irradiation position, the inverse of the value obtained using the above approximation was applied to each frame as the correction factor *k*_photon*t*_.

#### Reconstruction of leaf sinogram

**Fig. 9:**
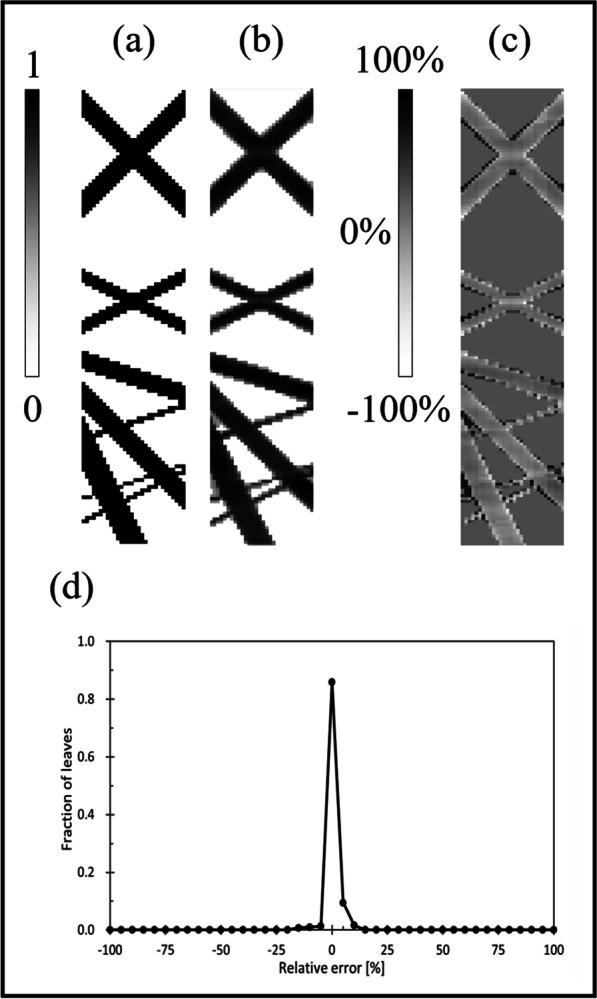
Reconstruction of the simple pattern sinogram. **a** Sinogram calculated by TPS, **b** Reconstructed sinogram. **c** Difference between the two sinograms, and the error of the LOT was − 2.2 ± 9.2%. **d** Histogram of errors and fractions of leaves, indicating that 85.8% of the leaves were detected within 5% of the error

The simple pattern sinogram resulted in the maximum Youden index when the threshold value was 23,200 pixels, with a sensitivity of 97.2% and a specificity of 98.0%. As shown in Fig. 5, the threshold value of 23,200 pixels corresponds to 2–4 open leaves. However, when only one leaf is open, the detected light per frame is 18,321 pixels. Therefore, using a threshold of 23,200 pixels may result in the recognition of "not open" when only one leaf is open. The IMRT sinogram resulted in a maximum Youden index of 98.1% sensitivity and 97.0% specificity at a threshold of 22,900 pixels. For the simple sinogram, the leaf could be misidentified as not open, even though it was open. The results of the reconstruction of the simple sinogram pattern are shown in Fig. 9. Figure 9a shows the sinogram calculated using TPS, Fig. 9b shows the reconstructed sinogram, and Fig. 9c shows the difference between the two sinograms. In Fig. 9c, the error of the LOT was considered as the average of the entire sinogram, which was − 2.2 ± 9.2%. In certain ROIs, the detected light exceeded the threshold, and the leaf was perceived as “open” even though the corresponding leaf was closed. This is because the light scattered from the irradiated area of the other open leaf pushed *q*_*n*_ above the threshold value at the edge of the irradiated field. Large errors are observed at the edges of the irradiation field. This may have been caused by the scattering of scintillation light on the sides of the PS.

**Fig. 10:**
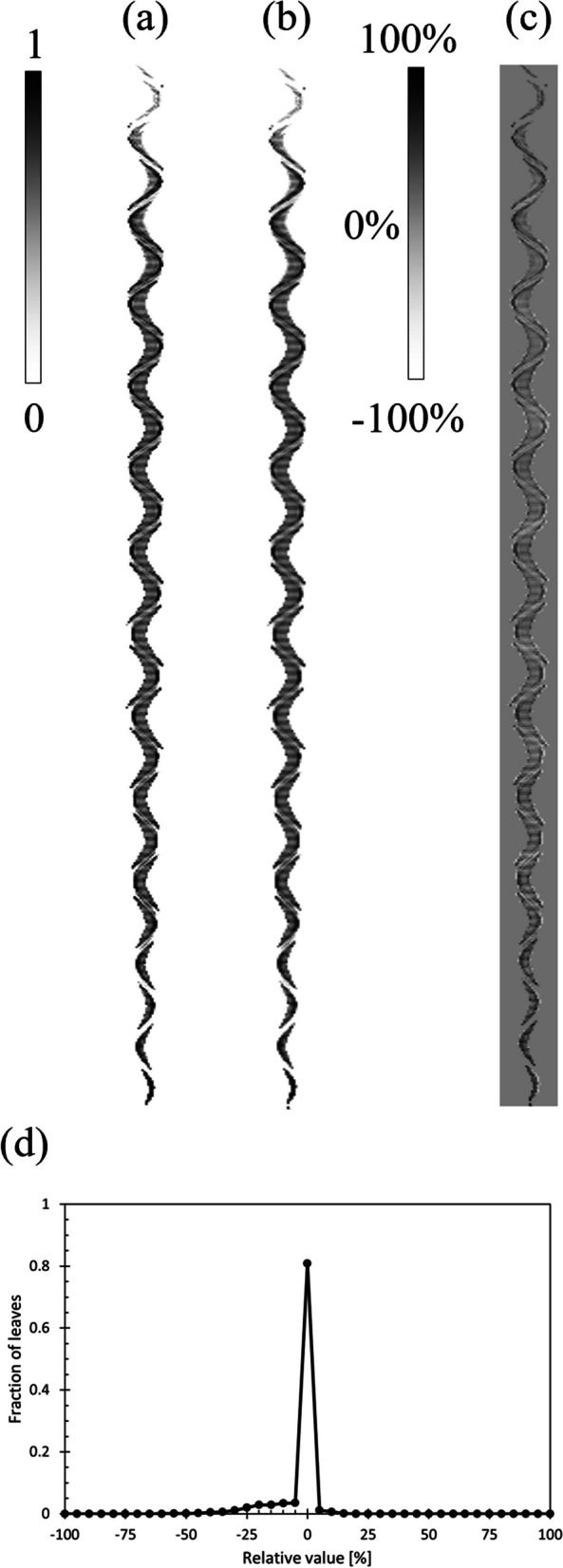
Reconstruction of the IMRT sinogram. **a** Sinogram calculated by TPS, **b** Reconstructed sinogram. **c** Difference between the two sinograms, and the error of the LOT was − 3.9 ± 7.8%. **d** Histogram of errors and fractions of leaves, indicating that 80.8% of the leaves were detected within 5% of the error

The results of the IMRT sinogram reconstruction are shown in Fig. 10. Figure 10a shows the sinogram calculated by TPS, Fig. 10b shows the reconstructed sinogram, and Fig. 10c shows the difference between the two sinograms. In Fig. 10c, the error of the LOT was considered as the average of the entire sinogram, which was − 3.9 ± 7.8%. As previously mentioned, large errors were observed at the edge of the irradiation field. However, the edge of the irradiation field in this sinogram was not near the surface of the PS. Because the relationship between the light intensity and LOT shown in Fig. 3 was obtained at the center of the irradiation field, it was not considered applicable to LOT because the light intensity decreased at the edge of the field.

### Measurement of dose distribution

#### Scattered coefficient

The optimization calculations yielded five PSF coefficients, the values of which are listed in Table 1. Scattering correction was performed by substituting the obtained counts into Eq. (3).

**Table 1:** Five coefficients for PSF post-optimization

a	b	c	σ_1_	σ_2_
0.709	0.212	0.095	0.236	11.7

**Fig. 11:**
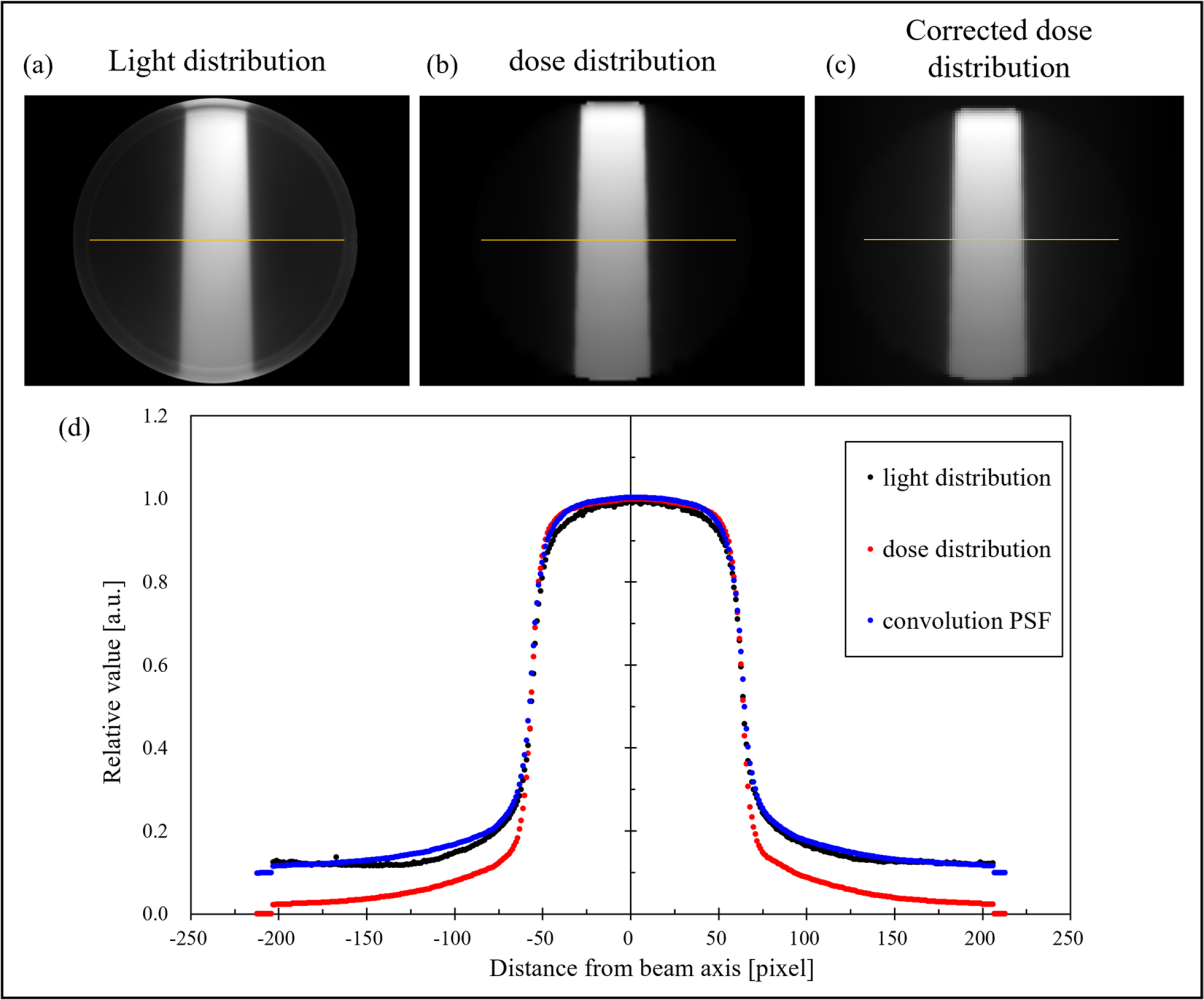
Scattered coefficient. **a** Light distribution, **b** Dose distribution calculated by TPS, and **c** Dose distribution after convolving PSF. **d** Profiles at the centers of the distributions in (**a**), (**b**), and (**c**), respectively

The images obtained after convolving the light distribution, dose distribution, and PSF are shown in Fig. 11a–c, respectively, and the profile through the PS center is shown in Fig. 11d. Figure 11d shows that before convolving the PSF, an error of approximately 8–10% at 60–200 pixels from the center was observed. However, after convolving the PSF, the difference improved by approximately 7–8%.

#### Measurement of the IMRT plan

**Fig. 12:**
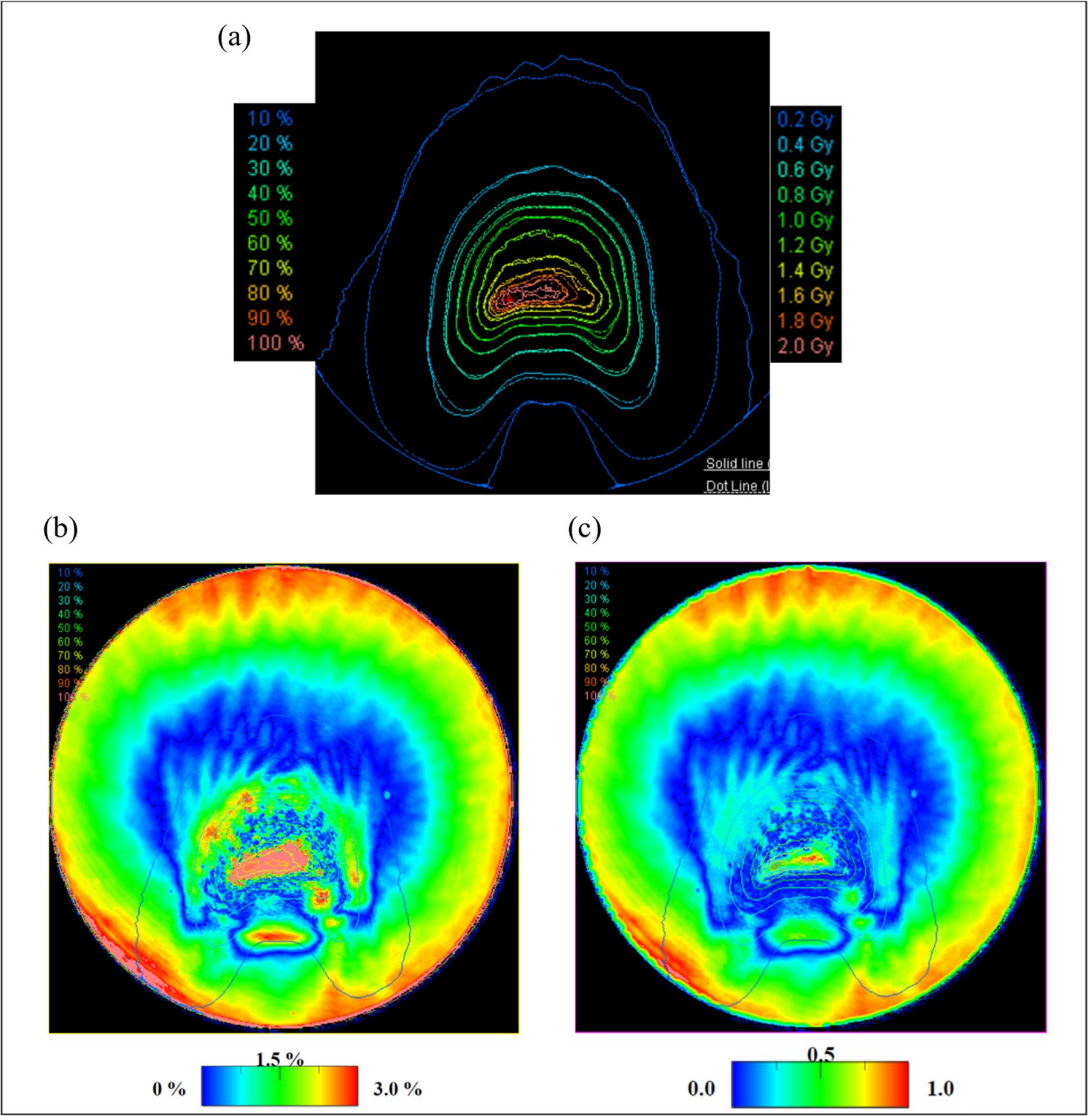
**a** Isodose curve with 100% being 2 Gy **b** Dose difference **c** Gamma analysis using the search criteria of 3% and 3 mm. The solid line of the isodose curve shows the dose distribution, and the dotted line shows the light distribution

The light distribution is similar to the dose distribution. Figure 12a shows the isodose curve with 100% being 2 Gy. The DD and the path rate of gamma analysis (3%, 3 mm) were calculated for the area above the 10% isodose curve. The DD was 1.38 ± 0.20% (mean ± standard deviation), and the pass rate was 99.8%. The dose and light distribution errors were larger toward the PS edge. This was due to the scattering of scintillation light on the sides of the PS. In addition, detecting the point where the X-rays were irradiated using the measuring instrument was difficult in this study. Therefore, we compared the 3D dose distribution of the IMRT plan with the light distribution by integrating the 3D dose distribution along the z-axis.

## Discussions

This study aims to develop a DQA system for simultaneous verification of dose distribution and mechanical movements in HT. In this study, we focused on binary MLC as a mechanical movement. Our results showed a LOT error of − 3.9 ± 7.8% in the reconstructed sinogram compared with the TPS. This outcome is consistent with the − 3.4 ± 8.0% accuracy reported in a previous study by Hashimoto et al. [19]. In a previous study, we demonstrated the feasibility of measuring binary MLC movements using a PS and a general-purpose camcorder. The high-speed operation of the binary MLC was captured by the camcorder at a high frame rate of 29.97 fps due to its speedy movement in the order of several tens of milliseconds. However, the camcorder records an 8-bit grayscale, which is inadequate for measuring the dose distribution, as required in this study. Thus, a cooled CCD camera with a frame rate of 8.2 fps and 16-bit grayscale recording was used in this study. Although the frame rate was lower than that of the camcorder, the measurements were performed within a comparable error range. The largest discrepancies were observed at the periphery of the irradiated field. This is likely because the relationship used to convert the scintillation light to LOT in Fig. 3 is established at the center of the irradiation field, and thus, it is less accurate at the edges of the irradiation field. Another source of error that may arise is the presence of scattered radiation that directly enters the CCD camera element, resulting in the temporary recording of noise within the collected images. This noise, known as transient noise [22], has been reported to be reducible through the use of a median filter. However, because filtering also alters the pixel values beyond the noise region, no filtration process was employed to alleviate the noise in this study, in which both the dose distribution and MLC movements were quantified using a single image. To resolve this issue, obtaining conversion equations for all MLC leaf configurations within the irradiation field was not feasible. For practical validation, evaluating the LOT error, specificity, and sensitivity would be adequate.

Although the results of the dose distribution measurements provided integral values, they were insufficient for determining a complete three-dimensional dose distribution. Efforts are underway to improve the detector for the identification of the irradiation position in the z-axis direction, thus enabling comparison with three-dimensional dose distributions and the reconstruction of sinograms corresponding to dynamic wedges. Additionally, the ability of this system to detect temporal changes in light distribution suggests its potential use in validating IMRT planning based on a four-dimensional dose distribution by analyzing the light distribution for each frame.

One of the key advantages of this study is the simplicity of the process flow from measurement to processing. The light intensity distribution and sinogram reconstruction are recorded in real-time using a CCD camera during irradiation, significantly reducing the time required for routine DQA. Nonetheless, the proposed detector system, which allows for the simultaneous measurement of dose distribution and equipment operation, is not currently available in existing systems, and it will enable a more detailed plan verification. It is important to acknowledge that the system does have limitations, particularly with respect to the reliability of its measurements for large-sized tumors due to the relatively lower accuracy of the PS in its periphery. In addition, the notable disparity observed outside the field can be attributed to scintillation light scattering within the PS. However, a 2D PSF correction was used in this study, further enhancement can be achieved through a 3D PSF correction, which will be a part of our future research endeavors.

## Conclusion

Examination of the dose distribution reveals a high degree of agreement between the light distribution obtained through the collection of the scintillation light, and that calculated using the TPS. The average DD above the 10% isodose curve was 1.38 ± 0.20%, and gamma analysis utilizing search criteria of 3% and 3 mm yielded a value of 99.8%. Examination of MLC opening demonstrated their ability to detect them through the reconstruction of sinograms from scintillation light. The developed system allows for the concurrent and accurate verification of both the dose distribution and MLC opening from the PS. Furthermore, the system has the potential to significantly reduce the time required for IMRT plan verification, which traditionally required several hours, thus enabling the provision of high-precision and safe radiation therapy to a greater number of patients. In the future, we plan to improve the detector so that the irradiation position in the z-axis direction can be specified, enabling the measurement of three-dimensional dose distribution.

## Data Availability

Not applicable.

## Abbreviations

IMRT: Intensity-modulated radiation therapy
VMAT: Volumetric modulated radiation therapy
HT: Helical tomotherapy
DQA: Delivery quality assurance
MLC: Multi-leaf collimator
PS: Plastic scintillator
3D: Three-dimensional
CCD camera: Charged coupled device camera
LOT: Leaf opening time
TPS: Treatment planning system
PSF: Point spread function
fps: Frame per second
ROI: Region of interest
DD: Dose difference
